# Complications Associated with Administration of Post-operative Weight-Based Enoxaparin in Orthopaedic Trauma Patients

**DOI:** 10.7759/cureus.21215

**Published:** 2022-01-13

**Authors:** Michael Booth, Owen Hamilton, Michelle Bramer, William Brooks, Michael Niemann

**Affiliations:** 1 Orthopaedics, West Virginia University, Morgantown, USA; 2 Orthopaedic Surgery, West Virginia University, Morgantown, USA

**Keywords:** weight based, orthopaedics, trauma, dvt prophylaxis, lovenox

## Abstract

Background

In orthopedic-specific patients, limited evidence exists in regard to prophylactic weight-based enoxaparin dosing in the obese population. We examined the clinical outcomes of administering weight-based enoxaparin to obese orthopedic trauma patients.

Methods

This retrospective study involved 679 patients who underwent orthopedic trauma surgery and were admitted from 1/2016 to 6/2020 at a single institution. Of those patients, 156 patients met our inclusion criteria. Inclusion criteria included BMI>35 kg/m^2^ and received weight-based enoxaparin post-operatively (defined as any singular dose >40 mg at any time). Blood transfusion, documented hematoma, deep vein thrombosis (DVT), and return visits to the OR after the administration of weight-based enoxaparin were the primary endpoints assessed. Age, BMI, weight, injury severity score (ISS), sex, post-operative time to the first dose of enoxaparin, the total daily dose of enoxaparin, operating room (OR) blood loss, OR time, patient co-morbidities, and pre/post-operative hemoglobin were evaluated for a potential relationship with the primary endpoints.

Results

One hundred and eighty-five surgeries were performed on a total of 156 patients. Thirty-six of the 185 (19%) surgeries required post-operative blood transfusion after weight-based enoxaparin was given. Higher ISS score, lower pre-operative hemoglobin, and lower post-operative hemoglobin were significant predictors of blood transfusion. Only increased post-operative time to the first dose of enoxaparin was significantly associated with DVT formation. Thirteen of the 156 patients (8.3%) had a post-operative hematoma after administration of enoxaparin, and four of the 13 patients required return to the OR for bleeding complications. ISS was the only significant predictor of post-operative hematoma formation.

Conclusion

Patients with a higher injury severity score are at an increased risk of adverse bleeding and may benefit from lower doses of enoxaparin administered earlier post-operatively.

## Introduction

Patients with traumatic injuries and obesity stand out amongst the highest-risk groups of developing venous thromboembolism (VTE) [1-7]. Obesity has been shown to be an independent risk factor for VTE in both men and women [8]. The risk of VTE in obese patients is estimated at twice that of non-obese patients and six times more likely in patients with a BMI of 35 kg/m^2^ or higher [9]. Obese individuals have an increased percentage of fat per kilogram of total body weight, and blood flow in adipose tissue is lower than lean body mass [10]. The difference of lean body mass and vascularization may affect drug kinetics [11]. Fixed doses of low molecular weight heparin (LMWH) in severely obese patients have higher failure rates of VTE prophylaxis [12]. Enoxaparin is a commonly used prophylactic medication used in the prevention of deep vein thrombosis (DVT) that potentiates antithrombin III to form a complex that irreversibly inactivates factor Xa [13]. Anticoagulant-based prophylaxis, including LMWHs, unfractionated heparin, and factor Xa inhibitors, reduces the relative risk of VTE by 45% to 63% in medically-ill patients [14-16]. Studies evaluating LMWH dosing in obese, trauma, and critically ill medical and surgical patients have found a strong negative correlation between weight and anti-Xa levels with fixed dosing and that weight-based dosing results in favorable anti-Xa activity [17-20]. Although there is good evidence to support the efficacy of weight-based dosing in obese patients [21-24], most of the studies have been using anti-factor Xa levels as the primary endpoint rather than using clinical driven outcomes [7].

One of the clinical observations we have seen in our post-operative, morbidly obese orthopedic patients is an increase in bleeding complications with weight-based enoxaparin dosing. A recent literature review of prophylactic enoxaparin dosing in obese orthopedic trauma patients yielded only low level studies reviewing the topic with no clear conclusion [24]. In morbidly obese, medically ill patients, the use of weight-based enoxaparin dosed at 0.5 mg/kg once daily is feasible and results in peak anti-Xa levels within or near recommended range for thromboprophylaxis without any evidence of excessive anti-Xa activity [7]. Our study explored possible relationships between weight-based anticoagulation dosing and adverse clinical outcomes.

## Materials and methods

Study design

Using our institution’s trauma literature database, we retrospectively reviewed patients who were admitted from 1/2016 to 6/2020 and who sustained an orthopedic injury requiring surgery. During that time period, 679 patients were admitted and required surgery. The study was approved by the West Virginia University Office of Research Integrity and Compliance (IRB protocol number 2005010366).

Inclusion and exclusion criteria

Inclusion criteria: BMI >35 kg/m^2^ and received weight-based enoxaparin post-operatively (defined as any singular dose >40 mg at any time). Exclusion criteria are the history of bleeding disorders, platelet count <100k, pregnancy or stroke within 14 days, and glomerular filtration rate <30 mL/min/1.72 m^2^. Of the 156 patients, a total of 185 surgeries were performed (21 of the 156 patients underwent multiple surgeries).

Outcomes

Blood transfusion, documented hematoma on ultrasound post-operatively, DVT, and return visits to the OR after the administration of weight-based enoxaparin were the primary endpoints assessed in these patients. DVT was included if not present prior to admission and confirmed by duplex ultrasound within three months after admission. Age, BMI, weight, injury severity score (ISS), sex, post-operative time to the first dose of enoxaparin (defined as the time in hours from exiting the operating room to the first dose of enoxaparin), the total daily dose of enoxaparin, calculated blood loss, OR time, patient co-morbidities, and pre and post-operative hemoglobin were evaluated for a potential relationship with the primary outcomes listed above.

Statistical analysis

Calculated blood loss was determined by using pre-operative and post-operative hemoglobin values while also considering BMI and gender using the Dibalsi et al. standard equation [25]. Co-morbidities such as diabetes, hypertension (HTN), chronic obstructive pulmonary disease (COPD), and smoking status were analyzed using Fisher’s testing to assess whether they would be predictive of patients developing a hematoma or requiring blood transfusion. T-test and one-way analysis of variance (ANOVA) was performed for testing a potential relationship between risk factors and need for post-operative blood transfusion.

## Results

One hundred and fifty-six patients, 76 male and 80 female, met our selection criteria, with a total of 185 surgeries being performed. One hundred and forty-nine (81%) of the surgeries did not result in post-operative blood transfusion after weight-based enoxaparin was administered. Thirty-six (19%) of the surgeries performed required post-operative blood transfusion after weight-based enoxaparin was given. Higher ISS (mean ISS transfused group 20.1 vs. 11.7 non-transfused group, p <0.0001), lower pre-operative hemoglobin (mean of 9.8 g/dl in transfused group vs. 12.3 g/dl in non-transfused, p <0.0001) and post-op hemoglobin (mean of 8.6 g/dl in transfused group vs. 10.8 g/dl in non-transfused group, p<0.0001) were significant predictors of post-operative blood transfusion after receiving weight-based enoxaparin. Age, BMI, weight, calculated blood loss, post-operative timing of enoxaparin, OR time, and the total daily dose of enoxaparin were not found to be significant predictors of blood transfusion post-operatively (Table 1). Diabetes, COPD, smoking status, and HTN were not predictive of post-operative blood transfusion.

**Table 1:** Differences between patients requiring a blood transfusion versus those who did not after receiving weight-based enoxaparin

Patient variables	Blood transfusion group (mean)	Non-transfused group (mean)	ANOVA Prob>F
Injury severity score	20.1	11.7	<0.0001
Pre-operative hemoglobin (g/dL)	9.8	12.3	<0.0001
Post-operative hemoglobin (g/dL)	8.6	10.8	<0.0001
Age (years)	49.8	47.6	0.4651
BMI (kg/m^2^)	46.7	46.7	0.9779
Weight (kg)	129.9	136.4	0.1823
Calculated blood loss (mL)	777.6	694.1	0.3676
Post-operative timing of first dose enoxaparin (hours)	21.3	29.0	0.3013
Total daily dose enoxaparin (mg)	129.2	124.5	0.5047
Operating room time (minutes)	223.7	195.8	0.0849

None of the 29 patients in the transfusion group experienced a DVT, while eight of the 127 patients (6.3%) in the non-transfusion group experienced a DVT within three months of discharge for an overall DVT rate of 5.1%. BMI, weight, ISS, calculated blood loss, pre/post-operative hemoglobin, the total daily dose of enoxaparin were not predictive of DVT (Table 2). Only increased post-operative time to the first dose of enoxaparin was significantly associated with DVT (mean of 65 hours from the end of surgery to the first dose of enoxaparinin DVT group vs. mean of 26 hours in no DVT group, p<0.01).

**Table 2:** Differences between patients who developed a DVT versus those who did not after receiving weight-based enoxaparin DVT - deep vein thrombosis

Patient variables	DVT	No DVT	ANOVA Prob>F
Injury severity score	13.1	13.3	0.9470
Pre-operative hemoglobin (g/dL)	12.7	11.7	0.2540
Post-operative hemoglobin (g/dL)	11.3	10.3	0.2082
Age (years)	59	48.5	0.08614
BMI (kg/m^2^)	47.0	46.7	0.9167
Weight (kg)	135.2	135.2	0.9979
Calculated blood loss (mL)	1048.0	697.2	0.0663
Post-operative timing of first dose enoxaparin (hours)	65.2	25.8	0.0057
Total daily dose enoxaparin (mg)	130	125	0.7246
Operating room time (minutes)	234.9	199.8	0.2653

Thirteen of the 156 patients (8.3%) had a clinically documented post-operative hematoma after administration of enoxaparin, and four of the 13 patients needed a return trip to the OR for bleeding complications after enoxaparin was administered. ISS (mean of 17.5 for hematoma group vs. 12.3 for the non-hematoma group, p<0.05) was the only significant predictor of post-operative hematoma formation while age, BMI, weight, OR time, the total daily dose of enoxaparin, and post-operative timing of first enoxaparin dose were not found to be significantly associated (Table 3). Diabetes, COPD, HTN, and smoking status were not associated with post-operative hematoma formation. 

**Table 3:** Differences between patients who developed a hematoma versus those who did not after receiving weight-based enoxaparin

Predictors of blood transfusion	Development of post-operative hematoma	No post-operative hematoma	ANOVA Prob>F
Injury severity score	17.5	12.3	0.0357
Pre-operative hemoglobin (g/dL)	11.4	12.1	0.3468
Post-operative hemoglobin (g/dL)	10.5	10.6	0.9518
Age (years)	52.5	48.2	0.3733
BMI (kg/m^2^)	47.4	46.9	0.8406
Weight (kg)	141.0	135.7	0.4839
Calculated blood loss (mL)	782.7	745.5	0.8075
Post-operative timing of first dose enoxaparin (hours)	23.5	30.7	0.5552
Total daily dose enoxaparin (mg)	133.8	124.6	0.4061
Operating room time (minutes)	217.6	199.1	0.4613

While weight-based dosing of enoxaparin may lead to adverse bleeding events, it is preventive of DVT formation. Patients with a higher injury severity score are at an increased risk of adverse bleeding events and may benefit from lower doses of enoxaparin administered at an earlier post-operative time. 

## Discussion

The results of our study show that there can be adverse bleeding events associated with the administration of weight-based enoxaparin. Thirteen of the 156 patients (8.3%) had a clinically documented hematoma with ultrasound after administration of weight-based enoxaparin, and four of the 13 patients required the return to the OR for bleeding complications. This is an important finding because there have been previous studies citing no adverse bleeding events associated with the initiation of weight-based enoxaparin [7,25]. The only significant predictor for an adverse bleeding event after receiving a dose of weight-based enoxaparin was an elevated ISS score. Therefore, in patients with higher ISS scores, one may consider non-weight-based dosing for DVT prophylaxis. This strategy is also supported by our findings that ISS was not predictive of post-operative DVT formation. Therefore it may be reasonable to begin for patients with a higher ISS score DVT prophylaxis earlier at a lower dose in order to adequately perform prophylaxis against DVT formation but prevent post-operative hematoma formation.

For example, none of the patients who required post-operative blood transfusions developed a DVT. However, it could be argued that these patients were over anti-coagulated, which necessitated blood transfusion or resulted in hematoma formation. The overall rate of patients developing DVT was 5.1%, which falls on the lower end of reported DVT rates (3-58%) after major orthopedic trauma regardless of BMI [2,5,6].

Much of the support for weight-based dosing of enoxaparin in obese patients is that standard dosing resulted in sub-therapeutic anti-Xa levels, which leads to an increased risk of DVT. In a retrospective review involving 792 trauma patients, Karcutskie et al. compared patients receiving a standard enoxaparin or heparin dose to an enoxaparin group that had their doses adjusted based on anti-Xa levels and found that there was no difference in VTE rates observed between those who reached the goal anti-Xa levels and those who did not [26,27]

There is also evidence supporting the idea that weight and BMI are not predictive of anti-Xa levels in patients receiving enoxaparin for DVT prophylaxis, which raises concerns about the reproducibility of using a weight-based approach [7,26]. Constantini et al. found little correlation between peak and trough levels of anti-Xa, which leads to more confusion about the consistent association for anti-Xa levels and DVT [28]. Proponents of weight-based DVT prophylaxis are supported by previous studies and case reports of previously under anti-coagulated patients who developed DVT or pulmonary embolism (PE). Imbalazano et al. reported a case of a 35-year-old man with a BMI of 32.9 kg/m^2 ^who developed a PE after being treated with only 40 mg of enoxaparin daily [29]. The conclusion of the study was that the patient was not adequately anti-coagulated, and the author suggested weight-based dosing of 0.5 mg/kg twice daily. 

This study is relevant because there is very limited evidence in the literature with regard to prophylactic enoxaparin dosing in obese orthopedic-specific patients [27]. The support for weight-based use of enoxaparin is largely based on bariatric studies that used therapeutic anti-Xa as a primary outcome to determine the success of the anticoagulation and did not cite any adverse bleeding events [7,20]. In 2008, the American College of Chest Physicians recommended weight-based dosing in obese medical and surgical patients, but their dosing was not delineated. This recommendation was based on a supporting study by Malinoski et al. that evaluated 54 critically ill trauma and surgical patients who received a fixed enoxaparin dose of 30 mg subQ BID for trough anti-Xa levels and then evaluated the incidence of VTE. The study found that 50% of patients had low anti-xA levels (defined as trough < 0.1 U/mL) and that low levels were associated with a significant increase in DVT rate (37% vs. 11%) [23].

This study has several limitations, including its retrospective nature and inability to account for all of the parameters that may account for post-operative bleeding and DVT formation. Also, our study does not differentiate based on the type of orthopedic surgery the patient underwent, which is important to reduce confounding variables. This may have predisposed the patient to more risk of DVT or an adverse bleeding event regardless of what treatment dose of enoxaparin the patient received. This is also a single-arm study, and we would have liked to compare traditional dosing of enoxaparin and our outcomes to the weight-based arm. Ideally, we would be able to prospectively evaluate patients with similar BMI, similar surgeries, and similar ISS scores and then assign them to weight-based enoxaparin versus standard dosing regimen. However, this would be incredibly difficult to match patients and account for different co-morbidities and medical considerations for the timing and administration of prophylactic DVT dosing. 

Overall, our study attempted to review how weight-based enoxaparin dosing is impacting orthopedic trauma patient outcomes in regards to DVT and adverse bleeding events. Despite previous studies not associating weight-based dosing with any adverse bleeding events, our study reveals that there are increased bleeding risks that may be associated with weight-based enoxaparin and one must balance the clear benefits of DVT prophylaxis with the potential risk of over anti-coagulating patients with weight-based dosing of enoxaparin. 

## Conclusions

Patients with a higher injury severity score are at an increased risk of adverse bleeding and may benefit from lower doses of enoxaparin administered earlier post-operatively.
